# A Comparison of Exposure Metrics for Traffic-Related Air Pollutants: Application to Epidemiology Studies in Detroit, Michigan

**DOI:** 10.3390/ijerph110909553

**Published:** 2014-09-15

**Authors:** Stuart Batterman, Janet Burke, Vlad Isakov, Toby Lewis, Bhramar Mukherjee, Thomas Robins

**Affiliations:** 1 Department of Environmental Health Sciences, School of Public Health, University of Michigan, 1420 Washington Heights, Ann Arbor, MI 48109, USA; E-Mail: trobins@umich.edu; 2 National Exposure Research Laboratory, U.S. Environmental Protection Agency, 109 T.W. Alexander Drive, Research Triangle Park, NC 27711, USA; E-Mails: burke.janet@epa.gov (J.K.); isakov.vlad@epa.gov (V.I.); 3 Department of Pediatric Pulmonary, Medical School, University of Michigan, 1500 East Medical Center Drive, Ann Arbor, MI 48109, USA; E-Mail: tobyl@med.umich.edu; 4 Department of Biostatistics, School of Public Health, University of Michigan, 1420 Washington Heights, Ann Arbor, MI 48109, USA; E-Mail: bhramar@umich.edu

**Keywords:** air pollution, dispersion modeling, epidemiology, exhaust, exposure misclassification, PM_2.5_, traffic, vehicle

## Abstract

Vehicles are major sources of air pollutant emissions, and individuals living near large roads endure high exposures and health risks associated with traffic-related air pollutants. Air pollution epidemiology, health risk, environmental justice, and transportation planning studies would all benefit from an improved understanding of the key information and metrics needed to assess exposures, as well as the strengths and limitations of alternate exposure metrics. This study develops and evaluates several metrics for characterizing exposure to traffic-related air pollutants for the 218 residential locations of participants in the NEXUS epidemiology study conducted in Detroit (MI, USA). Exposure metrics included proximity to major roads, traffic volume, vehicle mix, traffic density, vehicle exhaust emissions density, and pollutant concentrations predicted by dispersion models. Results presented for each metric include comparisons of exposure distributions, spatial variability, intraclass correlation, concordance and discordance rates, and overall strengths and limitations. While showing some agreement, the simple categorical and proximity classifications (e.g., high diesel/low diesel traffic roads and distance from major roads) do not reflect the range and overlap of exposures seen in the other metrics. Information provided by the traffic density metric, defined as the number of kilometers traveled (VKT) per day within a 300 m buffer around each home, was reasonably consistent with the more sophisticated metrics. Dispersion modeling provided spatially- and temporally-resolved concentrations, along with apportionments that separated concentrations due to traffic emissions and other sources. While several of the exposure metrics showed broad agreement, including traffic density, emissions density and modeled concentrations, these alternatives still produced exposure classifications that differed for a substantial fraction of study participants, e.g., from 20% to 50% of homes, depending on the metric, would be incorrectly classified into “low”, “medium” or “high” traffic exposure classes. These and other results suggest the potential for exposure misclassification and the need for refined and validated exposure metrics. While data and computational demands for dispersion modeling of traffic emissions are non-trivial concerns, once established, dispersion modeling systems can provide exposure information for both on- and near-road environments that would benefit future traffic-related assessments.

## 1. Introduction

Residential location and proximity to major roads have been widely used in analyses of exposures and adverse health effects that can result from traffic-related air pollutants, reflecting the elevated concentrations found near busy roads [1,2,3,4,5,6,7,8,9]. As an indicator or exposure surrogate, residential distance to roads, or more generally, residence location, is intended to reflect the portion of exposure received at home, which is generally the dominant share since most individuals spend the majority of their time at home [10]. Residence location or proximity to roads can be used as a surrogate exposure metric itself, or as an input to land use regression, dispersion, space-time (geostatistical), and hybrid models, which are designed to estimate ambient air concentrations and sometimes personal exposures [11,12,13,14,15].

Actual exposure for any particular individual will be the result of many factors, e.g., the amount of time spent indoors and outdoors, building and vehicle cabin air exchange rates, and breathing rates [12]. Unfortunately, direct measurement of traffic-related air pollutant exposure is rarely practicable due to cost and logistical issues [15]. Ambient air quality monitoring networks do not provide the spatial coverage needed to estimate near-road exposures [13], and personal, home or biomarker measurements rarely are feasible in large scale studies. Still, it remains important to improve exposure estimates that are used in epidemiology, health impact, environmental justice and other studies [1,14,16,17,18]. Improved estimates will minimize exposure misclassification that can bias results of epidemiology studies towards the null [19], incorrectly predict risks in health impact studies, and misidentify affected populations in environmental justice studies.

This paper explores alternate metrics for characterizing exposure to traffic-related air pollutants, including metrics based on proximity to major roads, traffic volume and density, traffic type, traffic emissions density, and pollutant concentrations from dispersion modeling. These metrics are being utilized in an ongoing epidemiology study investigating effects of diesel exhaust emissions on the respiratory health of asthmatic children in Detroit, MI, USA [20]. The evaluation of the exposure metrics presented in this paper includes a comparison of exposure distributions, spatial variability using maps coded by exposure group, intraclass correlations, and concordance rates.

## 2. Methods

### 2.1. Study Population

The Near-road EXposures and effects of Urban air pollutants Study (NEXUS) was designed to examine near-roadway exposures to air pollutants and respiratory health in children with asthma living near major roads in Detroit, MI. This community-based participatory research (CBPR) study was designed and conducted with community input and consent. Children with asthma or symptoms of asthma from 6 to 14 years of age were recruited on the basis of the proximity of their home to major roads in three traffic categories: high diesel/high traffic (HDHT), defined as homes within 175 m of roads with >6000 commercial vehicles/day (commercial annual average daily traffic; CAADT) and >90,000 total vehicles/day (annual average daily traffic; AADT); low diesel/high traffic (LDHT), defined as homes within 175 m of roads with >90,000 AADT and <4500 commercial vehicles/day; and low diesel/low traffic (LDLT) homes located >300 m from roads with >25,000 AADT and greater than 500 m from roads with >90,000 AADT. In the initial groups, the designation of commercial vehicles was used as a surrogate for diesel vehicles. Children in the LDLT group were drawn from the same neighborhoods and school catchment areas as the other groups, but lived further from high-traffic corridors, thus minimizing possible confounding from unmeasured neighborhood-associated covariates.

**Figure 1:**
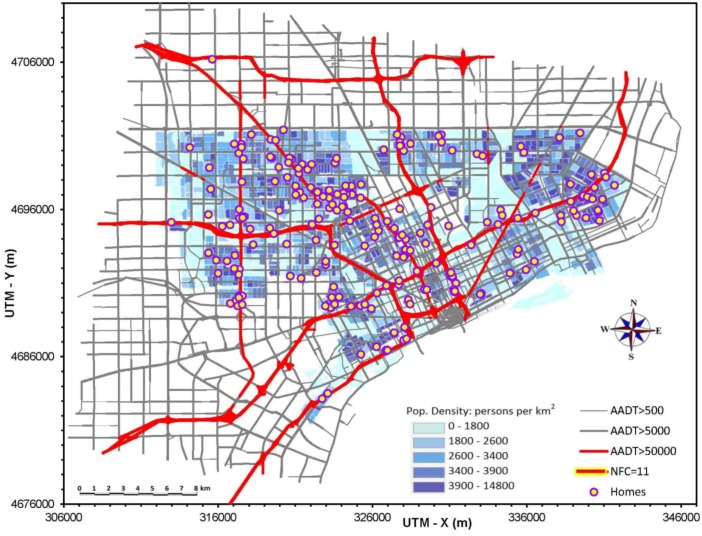
Map of modeled road network in study area, and locations of 218 homes of participants in NEXUS. Shaded area defines city of Detroit and population by Census Block group. Axis scales are Universal Traverse Mercator coordinates (m). AADT is annual average daily traffic (vehicles/day). Highlighted roads are National Functional Class 11, called high diesel/high traffic roads in NEXUS. Windsor, Canada (not shown), is immediately to the south-east.

Ultimately, 139 children were recruited and participated in the study from September 2010 to December 2012. Because a number of children moved during the study, a total of 218 residence locations were considered (Figure 1). The study population had approximately equal distribution across the three traffic categories. The population was primarily minority (non-Hispanic Blacks constituted 82% of the participants, Hispanics 8%, non-Hispanic Whites 4%, and other/multiracial 6%). Many households were poor (about one-third of families reported annual household incomes below $15,000).

### 2.2. Residential Proximity to Major Roadways

Because concentrations of traffic-related air pollutants rapidly decrease with distance from roads, considerable effort was spent to obtain accurate estimates of home locations and the distance to major road. Initially, to guide field staff in their recruitment efforts, candidate homes were identified using GIS-produced maps, which identified buffers within 200 m of selected highways and the street and house numbers of residences within these buffers. For the children recruited and enrolled into the study, the resulting 218 home locations were geocoded using three approaches. The first used a hand-held GPS device (60CS, Garmin International Inc., Olathe, KS, USA) operated by our technician who stood as close as possible to the residence’s front door. When the indicated accuracy was <10 m, the location was recorded on a data entry form and as a waypoint in the device’s memory. The calibration of the device was confirmed using several other GPS units. Second, online automated geocoding software provided by “Bing Maps” [21] was used by entering the number, street, city and ZIP code of each residence into this application, which returned latitude and longitude. The Bing Map estimates used the European Petroleum Survey Group (EPSG) code, a Mercator projection, and a spherical model of the earth [22]. The third geocoding estimate used the address locator in the online U.S. Streets Geocode Service in ArcGIS, ESRI ArcMap 10.0 (Redlands, CA, USA). This system uses a cascading sequence of geolocators starting with the Tele Atlas Address Points database, followed by the Tele Atlas Street Address Range database, 9-digit ZIP code, and then the 5-digit ZIP code locators [23].

All coordinates were converted to Universal Traverse Mercator (UTM) coordinates for use in subsequent analyses. If differences between the GPS and automated geocoding coordinates exceeded 100 m, data were checked, plotted, and if needed, our technician was sent out to confirm GPS coordinates a second (and sometimes a third) time, at which point all GPS measurements agreed. Final home locations are plotted in Figure 1. On average, the automated geocoding estimates diverged from GPS measurements at the NEXUS homes by an average 30 ± 23 m, although much larger errors were not infrequent, e.g., 75th and 95th percentile errors were ~50 and 75 m [24]. The bigger errors can represent a large fraction of the buffer width (200 m) used in the proximity metrics, suggesting that a fraction of homes geocoded using automated systems would be misclassified by such surrogate exposure metrics, especially since automated geocoding programs typically give larger errors than those found for Detroit [15,24].

Distances from each residence to the nearest roads and highways were determined using ESRI ArcMap (version 10.0) “NEAR” function within the Proximity toolset, the 2012 Topologically Integrated Geographic Encoding and Referencing (TIGER) 2012 road shape files, and the North American Datum (NAD) for 1983. A second measure of distance to the nearest highway was calculated using the link-based road network for the emissions inventory described next. This distance averaged 20 m greater than the TIGER estimate, representing the distance to the road centerline rather than to the road edge.

### 2.3. Road Network, Traffic Data and Emissions Inventory

The modeled road network used 9701 links (linear segments) to represent 3109 km of roads, which included all but the smaller and numerous local roads in the ~800 km^2^ study area (Figure 1). Major roads (e.g., freeways) were represented using separate links for each direction, large service roads, if any, and ramps. Road network data obtained from the Southeast Michigan Council of Governments (SEMCOG) included the locations, number of lanes, and roadway type (e.g., freeway, arterial). The road network extended at least 5 km beyond the locations of the NEXUS homes, and fully encompassed the city of Detroit (area of ~355 km^2^).

Hourly vehicle traffic volume and speed on each link were estimated using the SEMCOG Travel Demand Forecast Model, which used a TransCAD-based suite of applications, and temporal profiles for monthly, weekly and daily flows by vehicle class (e.g., heavy-duty diesel, light-duty gasoline). Where possible (e.g., for interstates), estimated vehicle flows were checked against observed traffic counts.

Estimates of total and diesel vehicle volumes on major roads formed the basis of the initial participant recruitment and the exposure classification. The initial grouping of roads (as HDHT, LDHT and LDLT) used 2006 and 2007 maps with AADT and CAADT flows prepared by the Michigan Department of Transportation [25]. As described earlier, subsequent analyses used state and regional data and a traffic demand model to derive AADT for each road link for the year 2010. Traffic on the major road closest to each HDHT and LDHT residence was estimated by summing AADT values on the corresponding road-links, which included at least two links (one for each direction) and occasionally additional links if the road split or if ramps added to the road’s traffic in the vicinity of the residence. At three homes, two major roads had similar proximity and both were counted in the AADT estimate. The corresponding number of lanes on these roads was summed as another measure of road size. Finally, the number of diesel vehicles on the closest major road was calculated as the product of the AADT (vehicles/day) and the fraction of diesel vehicles for the road type, which was 5.23% for “other freeways” (National Functional Class or NFC 12) and 9.18% for interstates (NFC 11). These fractions represent the sum of light-duty diesel trucks, heavy-duty diesel trucks, and heavy-duty diesel vehicles (LDDV, LDDT, HDDV) as derived using state-level data from the U.S. Federal Highway Administration [26], and the U.S. EPA Emission Inventory Improvement Program [27].

Primary mobile source emissions of particulate matter below 2.5 µm dia (PM_2.5_), oxides of nitrogen (NO_x_), carbon monoxide (CO) and other pollutants were estimated for each link on an hourly basis, thus producing a spatially and temporally resolved mobile source emissions inventory. The link-based inventory used emission factors representative of each vehicle class in the study area for the year 2010 calculated using the MOVES2010a model with inputs including the average speed and flow on each link, local vehicle mix and age distribution, ambient temperature, season, and road type [28,29]. Due to large uncertainties, particulate emissions for brake and pavement wear, and resuspension of dust were not included in the PM emissions estimates.

Emissions-based exposure metrics incorporate the quantity of traffic-related pollutant emissions released, providing an exposure metric that may be particularly relevant to policies addressing emission controls, transportation control measures, and other actions that directly affect emissions. Emissions density (g/day) was estimated as the daily vehicle exhaust emissions within a 300 m buffer around each home. PM_2.5_, NO_x_ and CO emissions were considered.

### 2.4. Concentration Estimates and Dispersion Modeling

We evaluated whether simple “box” models using the emissions density information discussed above could provide useful estimates of near-road pollutant concentrations. We assumed a wind speed u = 3.66 m/s (the long term Detroit average), a mixing height h = 100 m, and the average traffic-related PM_2.5_ emission rate Q in the 300 m buffer (*r =* 300 m) around high traffic homes. Concentrations were calculated as C = Q/(2 × u × r × h).

Hourly pollutant concentrations for the year 2010 were predicted at each home for three cases: annual average concentrations due to on-road exhaust emissions; the maximum 24-h concentration also due to on-road exhaust emissions, and the “total” annual average concentration due to on-road, non-road and background sources. Each case used the road-link emissions inventory for the Detroit area described above, the new RLINE dispersion model specifically designed for roadway emissions [30,31], and hourly meteorological data from the Detroit City airport processed by AERMET. RLINE is a steady-state Gaussian formulation for near-surface releases with dispersion parameters that can simulate low wind meander conditions. (The model is available from the U.S. Environmental Protection Agency [32]. The third case used a hybrid model system that integrated RLINE, the ERMOD model for area and point sources in the region (including Canada) using source locations, emission rates and other parameters from the 2008 National Emissions Inventory (NEI), and estimated regional (background) concentrations determined using the Community Multiscale Air Quality (CMAQ) model, observations from air quality monitoring networks in the region, and a space/time kriging model. As described elsewhere in this issue [31], this system is highly flexible, and model outputs can provide spatial and temporal patterns of air pollutants by source category.

Detailed descriptions and evaluations of RLINE and the other dispersion models have been presented elsewhere [30,31,33,34,35]. In Detroit, model results have been compared to ambient observations collected in both routine monitoring networks (AQS) and during the NEXUS intensive campaign. Compared to AQS data, 24-h average PM_2.5_ concentrations correlated well (0.78 < *r* < 0.94) with 2010 data collected at four PM_2.5_ monitoring sites in Detroit, and most predictions were within a factor of two of observations. NO_x_ concentrations predicted at the sole AQS monitoring site in Detroit reproduced morning and afternoon peaks but overpredicted the concentrations, likely due to contributions from regional sources since this site was several km from major highways. Compared to black carbon measured outside of 25 of the NEXUS homes and NO_x_ measured at 9 homes in (September–November) 2010, a pollutant often specific to traffic-related emissions, the model generally captured the magnitude and dynamics of observed concentrations, although concentrations were overpredicted or missed at some sites and some specific hours, likely due to uncertainty in hourly traffic activity and emissions at the road link level. Further description of the evaluation of the modeling system in the Detroit application is presented elsewhere [35].

### 2.5. Data Analysis

Descriptive analyses included graphs of distributions stratified by the original HDHT, LDHT and LDLT groups. Differences in means between the HDHT and LDHT groups were evaluated using *t*-tests, and difference in distributions for the same groups were evaluated using the non-parametric Mann-Whitney (MW) tests. (Sample size *n =* 96 in all cases for both tests). Comparisons between exposure metrics used Spearman’s and Kendall’s τ-b correlation coefficients. The latter correlation coefficient measures interclass agreement by considering the number of concordant pairs of observations minus the number of discordant pairs, expressed as the fraction of total pairs, and accounts for ties. Both are non-parametric measures that range from −1 to 1.

Additional measures of concordance/discordance rates were derived for exposures divided into “high”, “medium” and “low” categories to provide estimates potentially relevant to exposure misclassification, as discussed in the text. The first measure was defined as the percentage of homes identically classified after grouping each metric into tertiles (high, medium and low categories). Thus, 100% agreement indicates that each home is placed in the same tertile for the pair of metrics considered, while random assignment would be expected to yield 33% agreement. For distance, tertiles were reversed, *i.e*., the shortest distances (presumably the highest exposure) were placed in the third tertile. The skewness of several metrics produced non-uniform tertile ranges, *i.e*., for the number of lanes (0–6, 6–8 and 8–14), PM_2.5_ emissions (0–306, 306–1837, 1837–4027 g/day), 24-h peak (6–14, 14–18, 18–47 µg/m^3^) and annual average (0.8–1.6, 1.6–2.7, 2.7–9.4 µg/m^3^) PM_2.5_ concentrations. Thus, another concordance measure was used to examine agreement between “thirds,” defined using three evenly spaced bins between the metric’s minimum and a nominal maximum value, either the actual maximum or an adjusted value that provided at least 15 observations in the top third. This gave ranges for the distance metric of 3067–5025, 1534–3067 and 1–1534 m (reversed); 0–1300, 1300–2600 and >2600 g/day for PM_2.5_ emissions; 0.8–1.6, 1.6–2.7 and >2.7 µg/m^3^ for annual average PM_2.5_ concentrations; and 6–17, 17–27 and >27 µg/m^3^ for peak 24-h PM_2.5_ concentrations (Figure 3D, shown in section 3.2, illustrates differences between tertiles and thirds). Again, 100% agreement denotes that the pair of metrics placed each home at the same (low, medium or high) level.

## 3. Results

### 3.1. Grouping of Homes by Distance to Roads

By design, many of the children participating in NEXUS lived close to major roads. Most children in the high traffic groups (HDHT and LDHT) lived near one of two interstate highways or two State of Michigan highways: I-75 (*n =* 30 residence locations), I-94 (*n =* 20), M10 (*n =* 30), and M-39 (*n =* 16). A few children lived near I-96 (*n =* 3), which was classified as a “medium” diesel high traffic road and not considered further in the present analysis.

**Figure 2:**
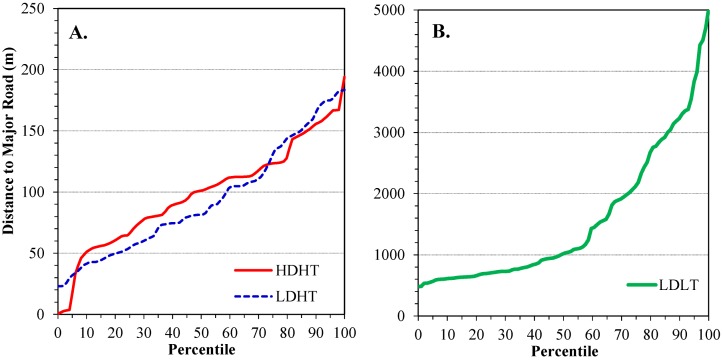
Distribution of distances of homes to major roads for the three traffic exposure groups. (**A**) HDHT (high diesel/high traffic) and LDHT (low diesel/high traffic) homes; (**B**) LDLT (low diesel/low traffic) homes. Based on GPS home location and road edge.

The distributions of distances from high traffic roads for the HDHT, LDHT and LDLT groups are shown in Figure 2. Means and distributions in the two high traffic groups did not differ (*t*-test: *p =* 0.641; MW test: *p =* 0.376), and the mean distance was 96 ± 45 m from the road edge. The distributions were rectangular (uniform) in nature, and one residence was as close as 1 m from the road edge (along I-75 in southwest Detroit). In contrast, distances in the LDLT group were much longer, averaging 1,562 ± 1,133 m (±standard deviation).

Among the HDHT and LDHT groups, distances from roads did not vary among the four major roads (Kruskal-Wallace test, *p =* 0.628), although the average distance for M-39 was 10 m less than those for the three other major roads. No general trends were noted for residence-road distances with respect to region of the city or distance from downtown.

Exposure metrics such as the proximity to roads, as well as metrics based on traffic activity and dispersion modeling that are discussed later, depend on the accuracy of geocoding for home and other locations, and the positional accuracy of the road network. The latter depends on many factors, including the representation of the road network, e.g., as road edges as used in the preceding analysis, or as links that represent the road centerline as used later with line source dispersion models. Both road shape files and road-link network generally closely matched detailed maps and photographs, however, discrepancies for road-links increased at road sections that were curved since link-based networks simplify geometry using a minimal number of linear segments. Such positional errors can lead to exposure misclassification, particularly for locations very near (e.g., within 20 to 50 m) of major roads.

### 3.2. Total and Diesel Traffic Metrics

**Figure 3:**
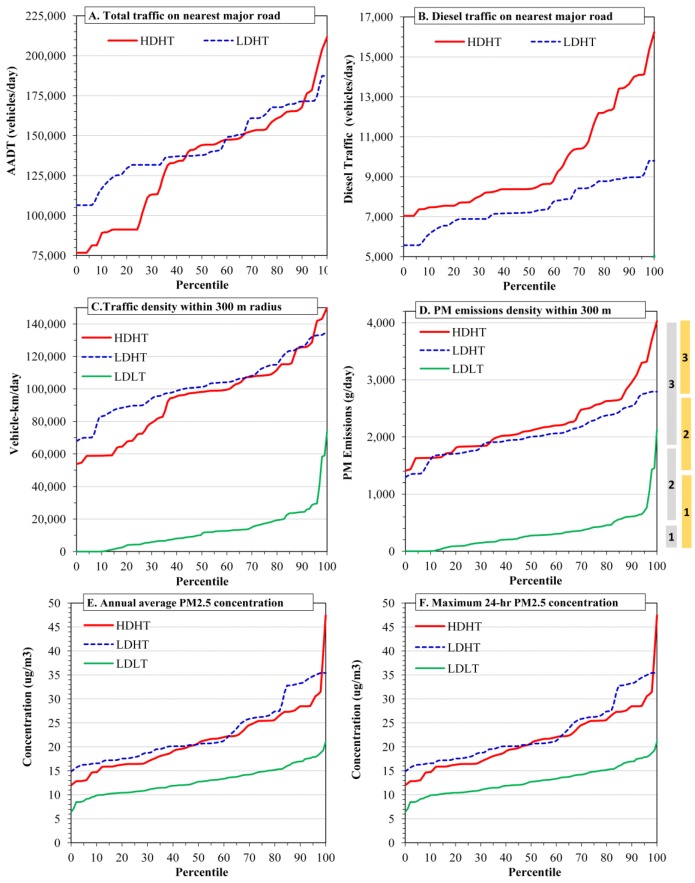
Distributions of selected exposure metrics coded by home group (HDHT = high diesel/high traffic; LDHT = low diesel/high traffic; LDLT = low diesel/low traffic). (**A**) Total traffic volume on major roads nearest home; (**B**) Diesel traffic volume on major roads nearest home; (**C**) Traffic density for roads within 300 m of home; (**D**) PM_2.5_ emissions density for roads within 300 m of home; (**E**) Annual average PM_2.5_ concentrations due to on-road emissions; (**F**) Maximum 24-h average PM_2.5_ concentrations due to on-road emissions. Panel D depicts differences between tertiles (in grey) and thirds (in orange).

**Figure 4:**
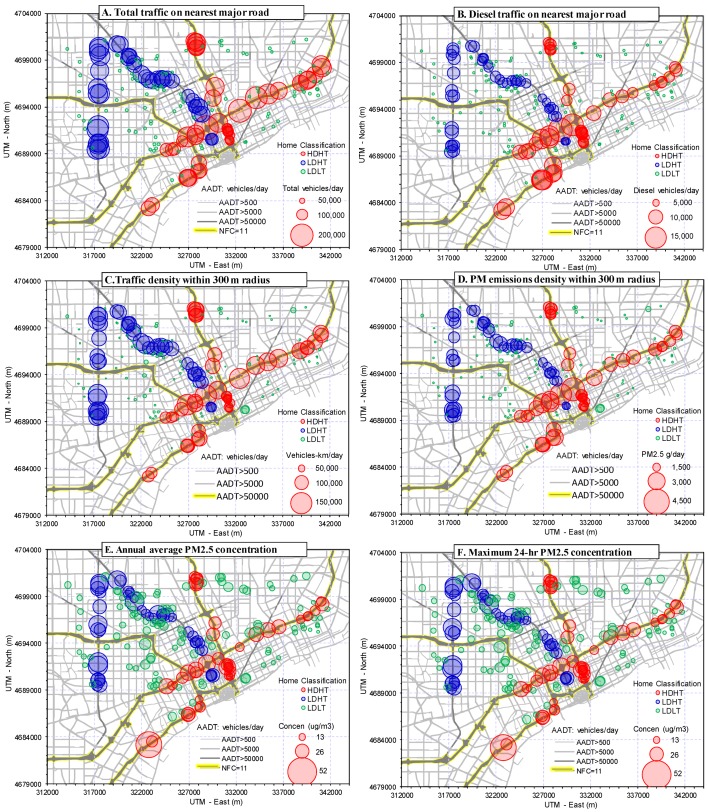
Depiction of selected exposure metrics mapped by home group (HDHT = high diesel/high traffic; LDHT = low diesel/high traffic; LDLT = low diesel/low traffic). (**A**) Total traffic volume on major roads nearest home; (**B**) Diesel traffic volume on major roads nearest home; (**C**) Traffic density for roads within 300 m of home. (**D**) PM_2.5_ emissions density for roads within 300 m of home; (**E**) Annual average PM_2.5_ concentrations due to on-road emissions; (**F**) Maximum 24-h average PM_2.5_ concentrations due to on-road emissions. In (**A**) and (**B**), LDLT homes are shown as green circles without coding for traffic volume.

The distributions of total and diesel traffic volume on major roads near the NEXUS homes are shown in Figure 3A,B and mapped in Figure 4A,B; descriptive statistics are in Table 1.

As anticipated, diesel vehicle traffic was highly correlated to the total traffic (*r =* 0.93), however, there are important differences, as discussed below. The maps show the clustering of high traffic homes along five major highways: HDHT homes fall mostly in the south and east along I-75 and I-94 (red circles, Figure 4A); LDHT homes are mostly in the north and west along M10 and M39 (blue circles). The LDLT homes (green circles) are distributed throughout the region.

**Table 1:** Statistics of AADT volume, diesel traffic volume, and number of lanes for the nearest highways at the high traffic (HDHT and LDHT) homes. Maximum values are affected by the three homes had equal distance to two highways (both were counted).

Statistic	All Traffic (Vehicles/Day)			Diesel Traffic (Vehicles/Day)			Number of Lanes		
	HDHT	LDHT	All	HDHT	LDHT	All	HDHT	LDHT	All
Average	133,737	143,965	138,638	9663	7529	8640	7.8	6.6	7.2
St. Dev.	34,962	21,614	29,634	2510	1130	2237	1.8	1.1	1.6
Minimum	76,723	106,508	76,723	7043	5570	5570	6	6	6
25th Percentile	94,202	131,718	124,586	7716	6889	7182	6	6	6
Median	144,013	137,845	140,722	8386	7209	8218	8	6	6
75th Percentile	153,576	162,808	160,968	11,297	8515	8974	9	7	8
95th Percentile	185,442	171,849	180,417	14,098	8988	13,711	11	9	10
Maximum	211,750	187,373	211,750	16,235	9800	16,235	14	10	14
Number	50	46	96	50	46	96	50	46	96

Considering the total traffic (AADT) near the high traffic homes, the average volume was 134,000 ± 30,000 vehicles/day and the average number of lanes was 7.2 ± 1.6. As noted above, three homes were equally near two major roads, including the home with the highest AADT, which was located at the I-94‒M-10 intersection (AADT = 212,000 vehicles/day, counting both roads); the two other homes were at the I-94‒I-96 and I-75‒I-96 junctions (178,000 and 134,000 vehicles/day, respectively). Traffic volumes at such junctions can be difficult to estimate given the numbers of ramps and highway segments involved. Otherwise, the traffic volume estimates were due to a single road. The AADT distributions for HDHT and LDHT homes were similar (*t*-test: *p =* 0.091; MW test: *p =* 0.262; Figure 3A), although the HDHT group had a greater range and its lowest tertile had 25,000 fewer vehicles/day. AADT had negligible correlation with the home’s distance to the road (*r =* −0.006). The maps suggest that each road had a wide range of traffic volumes, *i.e*., no apparent geographic bias.

Considering diesel traffic near the high traffic homes, the volume averaged 8640 ± 2240 vehicles/day, and eight homes had over 12,500 diesel vehicles/day. HDHT homes had an average of 2100 diesel vehicles/day more than LDHT homes, a statistically significant difference (*t*-test: *p <* 0.001; MW test, *p <* 0.001). Still, the distributions of HDHT and LDHT homes had considerable overlap, and roughly half of the two sets of homes had a comparable range of diesel volume (7000–9000 vehicles/day; Figure 3B). The highest diesel volume occurred for homes along I-75, while homes along the eastern portion of I-94 had lower diesel traffic volumes than other HDHT homes (Figure 4B). This resulted in part from reclassifying this section of I-94 to an “other highway” (NFC 12) designation rather than “interstate” in an attempt to better match the observed traffic classification. Diesel volume had negligible correlation with distance to the road (*r =* 0.001). Overall, estimates of diesel traffic are less certain than those for total traffic due to the variation in fleet mix and the limited classification data available.

By design, the LDHT homes were intended to have commercial annual average daily traffic (CADT) below 4500 vehicles/day. There are several reasons for differences between the initial groupings and those computed using the road-link network. First, not all diesel traffic is “commercial” (and *vice versa*). Based on the fleet composition data used, the bulk of diesel traffic on major roads are heavy duty diesel vehicles (HDDV), which represent 84% of the diesel volume on interstates, and 71% on “other highways”. The balance of diesel vehicles is contributed by light duty diesel vehicles (LDDV) and light duty diesel trucks (LDDT), which respectively represent 11% and 6% of the diesel vehicles on interstates, and 20% and 9% on other highways. With the exception of the 6% to 9% of the diesel vehicles classified as LDDVs, most LDDTs and all HDDVs form part of CADT. With the LDDV fraction removed, differences between the HDHT and LDHT categories slightly increase, e.g., the average volume of diesel traffic is 8700 ± 2,500 vehicles/day near the HDHT homes, and 6300 ± 950 vehicles/day near the LDHT homes. Second, CADT will include some gasoline-powered vehicles. However, the emissions inventory classifications do not correspond well to the AADT/CADT classifications. Third, the initial and road-link estimates of CADT used different data sources: the former depended on local measurements, while the latter used a mix of local, Michigan and national-level data.

### 3.3. Traffic Density Metric

A measure of traffic density, the number of vehicle-km traveled (VKT) per day within a 300 m radius of each home, was calculated using the road-link network. The average VKT/day was 94,200 ± 24,900 for HDHT homes, 102,200 ± 17,230 for LDHT homes, and 12,700 ± 12,400 for LDLT homes. Differences in traffic density between HDHT and LDHT homes approached statistical significance (*t*-test: *p =* 0.071; MW test, *p =* 0.117; Figure 3C). This metric varied considerably within each home group, e.g., the range spanned a factor of 2.8 for HDHT homes, and variation for homes along the same highway could be considerable, e.g., the southwest section of I-75 has several HDHT homes with low traffic density (Figure 4C). Of the LDLT homes, 4% (*n =* 4) had moderately high traffic density (41,000 to 74,000 VKT/day), overlapping or nearly overlapping the high traffic homes. Based on this exposure metric, the LDHT homes had slightly greater exposure than HDHT homes. 

For most high traffic homes, the largest share by far of the traffic density metric was contributed by interstates and other highways. This exposure metric can be very sensitive to the distance criterion since roads will drop out as the distance criterion (buffer size) decreases. For example, a 150 or 200 m buffer excluded the nearby highway for a few HDHT homes, dropping the KMT/day to nearly zero, despite large values obtained with the 300 m buffer. 

### 3.4. Emissions Density Metrics

Figure 3D and Figure 4D show the PM_2.5_ emissions density distributions and levels at each home. For PM_2.5_, the emissions density averaged 2,226 ± 568 g/day at HDHT homes, 2029 ± 379 at LDHT homes, and only 315 ± 320 at LDLT homes. The distributions of PM_2.5_ emissions near HDHT and LDHT homes appeared similar, although outliers caused results of the statistical tests to vary (*t*-test: *p =* 0.052; MW test: *p =* 0.153), e.g., a few HDHT homes near intersections of major roads (e.g., I-95 and I-75) had very high emissions. For the high traffic homes, nearby highways contributed the bulk of emissions. Emissions rates in the 300 m buffers around LDLT homes were much lower, although a few homes had emissions densities that overlapped those in the high traffic group.

Distributions and maps for NO_x_ and CO emissions density are shown in Supplemental Figures S1 and S2. Results for NO_x_ parallel the findings for PM_2.5_. However, results for CO diverged as HDHT homes tended to have lower CO emissions within the 300 m buffer, although statistical significance was not reached (*t*-test: *p =* 0.072; MW test: *p =* 0.093). Some of the higher levels were found for homes along M39 (in the western portion of the study region), due to the lower CO contributions from diesel vehicles and the dominance of CO emissions from much more numerous (and gasoline-powered) automobiles. Overall, the pattern for CO closely resembled the traffic density metric (Figure 3C and Figure 4C).

Emission-based metrics may be especially relevant for the traffic analysis zones (TAZ) commonly used by metropolitan planning organizations for various purposes, although TAZs may be relatively large relative to the spatial variability of traffic-related air pollutants. Emission-based metrics can be developed for the major road nearest point of interest (e.g., a home), for roads within a local area, or for buffers around points of interest, as presented here. This metric is pollutant-specific and thus can account for differences in fleet mix, technology and transportation control measures (TCMs) that can affect emissions.

### 3.5. Concentration Metrics

The “box” model using the average emissions density around high traffic homes for PM_2.5 _(2226 g/day) gave a predicted PM_2.5_ concentration of 0.12 µg/m^3^. This low estimate does not account for temporal variation in emission rates (e.g., rush-hour emissions are approximately twice the average rate) or meteorology (e.g., wind speeds near 1 m/s are not uncommon and mixing heights also vary). In addition, some homes had twice the average emission rate, and results will be sensitive to the buffer size and source configuration. The most significant limitation, however, is the validity of the fully mixed assumption near major roads where vertical dispersion of roadway emissions may not reach 100 m. As shown below, dispersion models designed for near-road applications yielded concentrations that were one to two orders of magnitude higher. Thus, while box models have been used at a city-wide or regional level [36], they do not appear useful for near-road applications. 

Dispersion modeling results for PM_2.5_ are summarized in Table 2, and concentration distributions and maps are shown in the bottom panels of Figure 3 and in Figure 4. Considering emissions from local traffic and annual average concentrations, PM_2.5_ levels at high traffic homes were 1.6 µg/m^3^ higher than those at LDLT homes, or about twice that at the LDLT homes. Considering 24-h peak concentrations, PM_2.5_ levels at several HDHT homes were estimated to exceed 40 µg/m^3^, which is high relative to PM_2.5_ standards. (Highly elevated concentrations at a few homes are shown as a sharp uptick in Figure 3E,F) (the U.S. National Ambient Air Quality Standards are currently 12 and 35 µg/m^3^ for annual and 24-h averages, respectively). The average 24-h peak across the high traffic homes was 11 µg/m^3^ more than levels at the LDLT homes, again about twice the level. Considering the “total” PM_2.5_ estimated by the hybrid model, regional sources were dominant, contributing about 12 µg/m^3^ on an annual average basis across the homes. Given the relatively high contribution of PM_2.5_ by regional sources, the spatial variability due to local sources was significantly reduced, e.g., PM_2.5_ levels at low and high traffic homes differed by only 10% to 20% (Distributions and maps of total PM_2.5_ are shown in the Supplemental Materials).

**Table 2:** Statistics of PM_2.5_ concentrations (µg/m^3^) predicted at home of NEXUS participants, classified by exposure group (HDHT = high diesel/high traffic; LDHT = low diesel/high traffic; LDLT = low diesel/low traffic). The hybrid annual average includes on-road, area, point and regional sources.

Statistic	Onroad, Annual Average			Onroad, 24-hr Peak			Hybrid, Annual Average		
	HDHT	LDHT	LDLT	HDHT	LDHT	LDLT	HDHT	LDHT	LDLT
Average	3.3	3.2	1.5	21.4	22.8	12.9	15.6	15.6	13.9
St. Dev.	1.2	1.0	0.4	6.3	6.2	2.9	1.4	1.5	1.3
Minimum	2.2	2.1	0.8	12.0	14.9	6.4	13.3	13.7	12.1
25th Percentile	2.7	2.4	1.3	16.4	17.9	10.7	14.6	14.4	13.0
Median	2.9	3.0	1.5	20.8	20.6	12.7	15.4	15.3	13.6
75th Percentile	3.8	3.6	1.6	25.4	26.2	14.8	16.2	16.3	14.4
95th Percentile	4.8	4.8	2.4	29.6	34.7	17.9	18.2	18.6	16.5
Maximum	9.4	6.4	3.3	47.4	35.4	21.1	20.7	19.2	19.7
Number	50	46	102	50	46	102	50	46	102

The distributions of annual average PM_2.5_ concentrations at HDHT and LDHT homes due to local traffic were very similar (*t*-test: *p =* 0.637; MW test: *p =* 0.523, Figure 3E and Figure S3); peak 24-h concentrations tended to be slightly but not statistically higher at the LDHT homes (*t*-test: *p =* 0.251; MW test: *p =* 0.235; Figure 3E,F). The highest prediction, considered an outlier since it considerably exceeded any other prediction, occurred at a home very near I-75. Excluding this point, concentrations due to local traffic varied by about 3-fold in each exposure group.

Unlike the metrics discussed earlier, dispersion modeling results are expressed as concentrations that permit direct and meaningful comparisons to air quality standards, monitoring data and other studies. For example, a recent application of a hybrid dispersion model in London, England showed annual average PM_2.5_ contributions from 1 to 5 µg/m^3^ due to local vehicle exhaust (depending on site), and about 11 µg/m^3^ due to other local and regional sources [11]. Both the traffic and regional PM_2.5_ contributions were very similar to those predicted for Detroit. While an intercity comparison incorporating meteorology and spatial factors is beyond the present scope, the volume of traffic and the number of large and high emitting diesel trucks on Detroit’s highways may produce PM_2.5_ concentrations that are comparable to those in London, despite Detroit’s smaller size and lower fraction of diesel vehicles.

### 3.6. Comparison of Exposure Metrics

Table 3 compares the exposure metrics using Spearman’s and Kendall’s τ-b correlation coefficients. To include the original categorical proximity classifications in this analysis, LDLT, LDHT and HDHT groups were coded two ways: as 1, 2 and 3 respectively in the “Group1” variable, with the assumption that exposures were ranked as HDHT > LDHT > LDLT; and as 1, 2 and 2 in the “Group2” variable with the assumption that exposures were HDHT ≈ LDHT > LDLT, as suggested by most metrics. Table 3 colors the higher (>0.6 and >0.8, absolute value) correlation coefficients. For the Spearman coefficients, absolute values above 0.17 (*n =* 96 for comparisons involving only high traffic groups) or 0.12 (*n =* 198 to 218 for other comparisons) are statistically significant (*p <* 0.05, 1-sided test). While the measures have some differences, e.g., the Spearman coefficients are 3% to 23% higher than the Kendall τ-b coefficients and contrasts in Kendall’s τ-b coefficients tend to be larger, similar patterns emerge.

The original proximity classifications (“Group1” and “Group2”) are related to distance to the nearest highway (defining variables), to traffic density (VKT/day in the 300 m buffer around each home), and to PM_2.5_ and CO emissions density metrics using the same buffers. While maximum 24-h and annual average PM_2.5_ concentrations from local traffic were closely correlated, predicted PM_2.5_ concentrations had only modest agreement with most of the other exposure metrics, although correlations with the traffic density and distance metrics might be viewed as reasonable.

Differences among the metrics can occur for a number of reasons. Metrics that depended on a single road, *i.e*., the original groups, distance to, traffic volume on, and number of lanes on the nearest major road, fared poorly in comparisons since these metrics excluded the influence of other roads, among other reasons. Still, these simple metrics have some value. Second, traffic density and emissions density are very strongly related, and may yield equivalent spatial patterns. Third, predicted concentrations (e.g., annual average and 24-h maxima) were not as strongly related to the two density metrics, most likely a result of the dispersion modeling accounting for meteorological influences, e.g., directional effects. Fourth, while concentrations of different pollutants and concentrations using different averaging times and statistics (e.g., average *vs.* peak) show differences, the long term spatial patterns are relatively similar and a single metric may be sufficient for many applications. (In contrast, short-term patterns, e.g., daily levels, will vary considerably due to both the influence of meteorology and traffic patterns.) In health effects studies such as NEXUS, it is also useful to understand the relative ranking of exposures, discussed next.

Table 4 presents results of the concordance analysis related to exposure misclassification (assuming that one of the metrics in the pairwise comparison reflects the true exposure). Metrics with higher (>60% and >80%) agreement are highlighted. The concordance measures estimate the misclassification relevant for comparison to the three original groups (HDHT, LDHT, LDLT). As discussed next, concordance measures can be sensitive to the metric’s distribution and the bin cutoffs used.

**Table 3:** Comparison of exposure metrics at the NEXUS homes using Spearman (top) and Kendall τ-b correlation coefficients. Shaded numbers show absolute value of correlations above 0.6 and 0.8. Variables: Group1 = LDLT, LDHT and HDHT assigned 1, 2, 3, respectively; Group2 = LDLT, LDHT and HDHT assigned 1, 2, 2, respectively; Distance = distance to nearest major highway (LDHT and HDHT only); AADT = traffic volume on nearest major road (LDHT and HDHT only); Lanes = number of lanes on nearest major road (LDHT and HDHT only); Diesel = diesel vehicles volume on nearest major road (LDHT and HDHT only); VKT = traffic density as vehicles-km-traveled/day in 300 m buffer around home; PMemis = PM_2.5_ emissions in 300 m buffer; COemis = CO emissions in 300 buffer; PMave = 2010 annual average PM_2.5_ concentration due to local traffic; PMmax = 2010 maximum 24-h average PM_2.5_ concentration from local traffic; PMtot = 2010 annual average PM_2.5_ concentration from all sources. Sample size is *n =* 218, except for comparisons involving AADT, Lanes, and Diesel metrics where *n =* 116 since only high traffic homes are considered. (a) not calculated due to variable definition.

Type and Metric	Group1	Group2	Distance	AADT	Lanes	Diesel	VKT	PMemis	COemis	PMave	PMmax	PMtot
	(Group)	(Group)	(m)	(veh/day)	(no)	(veh/day)	(km/day)	(g/day)	(g/day)	(ug/m^3^)	(ug/m^3^)	(ug/m^3^)
**Spearman correlation coefficients**												
Group1	1.00											
Group2	0.95	1.00										
Distance	−0.81	−0.87	1.00									
AADT	−0.12	(a)	0.06	1.00								
Lanes	0.39	(a)	0.31	−0.08	1.00							
Diesel	0.47	(a)	−0.02	0.66	0.12	1.00						
VKT	0.80	0.86	−0.66	0.50	−0.43	0.26	1.00					
PMemis	0.83	0.85	−0.66	0.36	−0.33	0.47	0.98	1.00				
COemis	0.79	0.86	−0.66	0.48	−0.43	0.25	1.00	0.98	1.00			
PMave	0.78	0.82	−0.75	0.15	−0.24	0.23	0.78	0.78	0.78	1.00		
PMmax	0.70	0.76	−0.71	0.35	−0.23	0.27	0.74	0.73	0.75	0.90	1.00	
PMtot	0.56	0.59	−0.57	0.13	−0.08	0.17	0.55	0.54	0.55	0.74	0.71	1.00
**Kendall Tau-B coefficients matrix**												
Group1	1.00											
Group2	0.90	1.00										
Distance	−0.62	−0.71	1.00									
AADT	−0.10	(a)	0.04	1.00								
Lanes	0.36	(a)	0.23	−0.07	1.00							
Diesel	0.39	(a)	−0.02	0.60	0.09	1.00						
VKT	0.61	0.71	−0.42	0.42	−0.34	0.19	1.00					
PMemis	0.66	0.70	−0.42	0.28	−0.26	0.36	0.90	1.00				
COemis	0.60	0.70	−0.41	0.40	−0.34	0.18	0.98	0.91	1.00			
PMave	0.62	0.67	−0.58	0.12	−0.18	0.16	0.55	0.56	0.55	1.00		
PMmax	0.54	0.62	−0.52	0.27	−0.18	0.19	0.53	0.52	0.53	0.74	1.00	
PMtot	0.44	0.49	−0.40	0.09	−0.06	0.12	0.38	0.37	0.38	0.56	0.52	1.00

**Table 4:** Comparison of exposure metrics at the NEXUS homes showing concordance with classifications using tertiles and “thirds”. Shaded numbers show percentage agreement above 60 and 80%. Variables and sample size are defined in Table 3.

Type and Metric	Group1	Group2	Distance	AADT	Lanes	Diesel	VKT	PMemis	COemis	PMave	PMmax	PMtot
	(group)	(group)	(m)	(veh/day)	(no)	(veh/day)	(km/day)	(g/day)	(g/day)	(ug/m^3^)	(ug/m^3^)	(ug/m^3^)
**Agreement among tertiles (percent)**												
Group1	100											
Group2	75	100										
Distance	58	44	100									
AADT	28	36	33	100								
Lanes	24	26	18	38	100							
Diesel	33	39	27	56	34	100						
VKT	52	45	49	34	19	37	100					
PMemis	56	45	49	37	17	43	89	100				
COemis	53	45	48	33	21	35	96	90	100			
PMave	60	51	66	32	20	34	58	61	59	100		
PMmax	54	45	62	41	18	35	58	56	57	75	100	
PMtot	55	52	58	42	22	36	51	50	50	64	64	100
**Agreement with thirds (percent)**												
Group1	100											
Group2	75	100										
Distance	31	7	100									
AADT	45	71	16	100								
Lanes	15	33	15	34	100							
Diesel	53	76	21	67	40	100						
VKT	70	76	28	35	28	42	100					
PMemis	76	90	14	60	31	74	80	100				
COemis	70	73	30	33	28	38	97	78	100			
PMave	64	80	12	47	33	50	70	77	69	100		
PMmax	65	76	15	60	30	48	68	76	66	77	100	
PMtot	56	68	13	44	39	43	61	62	59	72	67	100

The original proximity classification for NEXUS homes (“Group1” in Table 4) matched the tertiles and thirds groupings for PM_2.5_ concentrations predicted by the dispersion model for 54% to 65% of homes. Considering only the low and high traffic categories (“Group2”), the percentage of homes that matched PM_2.5_ concentration tertiles was lower (45% to 52%), but higher for thirds (60% to 80%). The same five metrics that were highly correlated (traffic density, emissions density for CO and PM_2.5_, annual average and peak 24-h average PM_2.5_ concentrations from traffic, Table 3), and sometimes the total PM_2.5_ (including all sources) had higher agreement, e.g., 50% to 96% using tertiles, and 59% to 97% using thirds. Very high concordance was found (and expected) for traffic density and CO emissions density. Overall, the concordance measures in Table 4 and the correlations in Table 3 produced similar patterns among the metrics. While over half of the homes were similarly placed into “low”, “medium” or “high” traffic exposure classes using either the original groups or one of the other metrics discussed, 20% to 50% of homes, depending on the metric, were categorized differently.

We also examined rates of “severe” discordance using both tertiles and third, but considering homes placed in the high exposure group by one metric, but in the low exposure group by the second metric (Table S1). Paralleling the analysis above, severe discordance rates among five more comprehensive metrics (traffic density, CO and PM_2.5_ emissions densities, and annual average and 24-h peak concentration) were low (0%–6%).

However, rates using total or diesel traffic volume on the nearest major road with these five metrics were 11% to 22% using tertiles, and 43% to 52% using thirds. Severe discordance rates using the simple distance-to-highway metric compared to the five metrics were also low (1%–3% using tertiles), meaning that few LDLT homes were placed in the upper tertiles of the five metrics and few HDHT or LDHT homes were placed in low tertiles of the same metrics. In addition to reinforcing earlier conclusions, this analysis indicates severe discordance is rare among exposure metrics using traffic density, emissions density, or concentrations.

## 4. Discussion

Table 5 summarizes the exposure metrics, describing their strengths, limitations, data requirements, and key results in NEXUS. Each metric provides different information (noted in the table and not repeated here). Several additional points are noteworthy. First, traffic-related exposures should be viewed as a continuum, and exposure groups are not “homogeneous.” For example, most of exposure metrics for the NEXUS homes in each of the three initial groups (LDLT, LDHT and HDHT) typically spanned a 3-fold range, with considerable overlap between groups. Second, exposures result from multiple emission sources, and concentrations depend on source characteristics (e.g., emission rate) and dispersion (e.g., wind direction and stability), factors that vary in time. While an emphasis on nearby sources (e.g., major roads) makes analyses more tractable, such simplifying assumptions can be inaccurate, and the choice of a distance cut-off is arbitrary. As examples, concentrations of traffic-related pollutants at NEXUS homes near major highways varied greatly from day-to-day, and homes that were distant (>500 m) from highways sometimes still received considerable levels of traffic-related pollutants. Third, the minimum information needed to evaluate spatial variability (typically using the long term average) of traffic-related exposure includes the distance-to-road and traffic volume for the larger and nearby roads.

The suggested minimum information, distance-to-road and traffic volume, can be expressed in the relatively simple traffic density metric, which correlated reasonably well with predicted PM_2.5_ concentrations (*r* = 0.78 for annual averages, *r* = 0.74 for 24-h peaks). Traffic density has been identified as one of the strongest predictor variables in recent land-use regression (LUR) models of traffic related air pollutants [37]. However, traffic density metrics do not provide concentrations and, even if incorporated into LUR models, results may be limited in terms of comparability across cities and time [38,39]. The dispersion models provided spatial and temporal concentration predictions as well as source apportionment information, e.g., the PM_2.5_ share due to traffic. While data and computationally intensive, such models have a strong physical basis and also are amenable to forecasting and scenario analysis [14].

**Table 5:** Summary of metrics used for exposure to traffic-related air pollutants in NEXUS.

Type	Exposure Metricas Defined for NEXUS	Strengths	Limitations	Results in Detroit
1. Distance to major road	Distance from home to road edge, and distance from home to road centerline, using GPS home location.	Simple to construct. Low data needs. Can potentially distinguish roads with varying traffic volume, vehicle mix, or other characteristics.	Distance limit used as cutoffs for classifying homes/receptors is arbitrary. May not consider traffic volume, vehicle mix, and other factors. Sensitivity to distance calculation, e.g., using road edge or centerline.	HDHT and LDHT roads had comparable distances to homes. LDLT distances considerably exceeded HDHT and LDHT groups, by design and recruitment approach.
2. Total traffic volume on nearby roads	AADT roads within 200 m of homes, using nearest road edge and GPS home location.	Relatively simple to construct. Reasonably good volume estimates on major roads. Can select period of day, e.g., rush-hour.	Traffic volume estimates needed. Distance criterion used to determine road is arbitrary. Does not provide metric for low traffic groups.	HDHT and LDHT groups largely indistinguishable. HDHT group had considerable range.
3. Diesel (or truck or commercial) traffic volume on nearby roads	Roads within 200 m of homes using road edge and GPS home location.	Relatively simple to construct. May relate to PM emissions from diesel traffic. Can select period of day.	Difficult to estimate diesel traffic volume accurately. Does not account for type of diesel vehicles and emissions. Otherwise as 2 above.	HDHT and LDHT groups were largely indistinguish-able. HDHT group had roughly 10%-20% higher diesel volumes than LDHT group, but about 2/3 of the values overlapped.
4. Local traffic density	AADT on road segments with 300 m distance (buffer) around each home, based on distance to road centerline, GPS home location, and traffic-demand model estimates of AADT.	Includes local traffic emissions that might affect receptor. Result (VKT/day) is easily interpretable and possibly generalizable. Large range across sites. Can be applied to irregular shaped sources and receptors. Can select period of day. Relevant to traffic analysis zones used by planners.	Moderately high data needs. Computationally intensive. Sensitive to distance criterion, which is somewhat arbitrary. Uncertainty of traffic estimates on all but major roads. Excludes smaller roads.	LDHT group had slightly greater exposure than the HDHT group. All but a few LDLT homes had low values.
5. Emissions on local roads	As 4 above with addition of annual average road-link emissions estimates for PM_2.5_, NO_x_ and CO.	Incorporates vehicle emissions of pollutants of interest. Reflects vehicle mix on roads. Also as 4 above.	Results depend on pollutant, to an extent. High data needs. Computationally intensive. Difficult to estimate emissions accurately.	For PM_2.5_ and NO_x_, HDHT had slightly higher exposure than LDLT. For CO, results are reversed but very similar All but a few LDLT homes had much lower values.
6. Pollutant concentration predictions	PM_2.5_ predictions at homes used road-link emissions inventory and RLINE dispersion model; area and point sources using AERMOD and regional sources handled using CMAQ and kriging interpolations of monitoring data.	Incorporates effects of emissions, meteorology, and location in physically-based approach. Quantifies and apportions concentrations due to each sources, e.g., traffic. Can be derived for specific periods of day, season or year, e.g., daily predictions at rush hour periods. Inter-study comparisons are possible and meaningful.	Results depend on pollutant, averaging time, and statistic. High data needs. Computationally intensive. Uncertainty not well characterized. Results potentially sensitive to many factors, including home placement.	For PM_2.5_, HDHT and LDHT distributions were similar although some dependence on averaging time and statistic. PM_2.5_ contributions from local traffic at HDHT and LDHT homes were about twice those at LDLT homes. Regional sources provide much (80%) of total PM_2.5_, but smaller contributions of NO_x_ and CO.

This paper has focused on exposure metrics for on-road vehicle emissions. Results for other pollutants and other locations will depend on the pollutant itself, the relative significance of on-road, area, point and regional emission sources, the locations of emission sources with respect to residence and other locations frequented by the population, the prevailing meteorology and terrain. In Detroit and many other cities, results expressed in Table 5 would likely be similar for NO_2_, carbon monoxide, elemental carbon, PAHs, and ultrafine PM given the significance of on-road emissions in urban areas. However, results for volatile organic compounds (VOCs) may differ given the large share of emissions occurring during refueling and at other non-road locations, while results for PM_2.5_ may be dominated by background levels and secondary formation. As noted in the table, these issues can be addressed by dispersion models, but these differences will not be captured by simpler metrics like proximity to roads and traffic density. Further differences may exist between regions and countries. In the U.S., for example, the vehicle mix is dominated by spark ignition (gasoline) engines that have low PM emissions, and most vehicles have catalytic and other controls that reduce gaseous emissions, however, most cities are surrounded and bisected by high traffic freeways. In Europe, in contrast, cities tend to have fewer freeways, thus traffic (and emissions) may be somewhat more evenly distributed, but the European fleet has a large fraction of diesel vehicles with higher PM_2.5_ emissions, and emissions of other pollutants historically have been less well controlled. While this can increase pollutant levels in urban areas and strengthen linkages between proximity, traffic intensity and pollutant concentrations, background levels can be high for pollutants like PM_2.5_. Given that land-use regression models have performed well in both U.S. and Europe for several pollutants, relationships between the simpler exposure metrics and pollutant concentrations in U.S. and European cities may be, in general, comparable to those in Detroit, although the reasons for the agreement may vary.

## 5. Conclusions

Surrogate exposure metrics for traffic-related air pollution exposures developed and compared in this study include proximity to highways, traffic volume, traffic density, number of lanes, emissions density, and concentration predictions from dispersion models. These metrics were critiqued individually and collectively, focusing on results obtained at the 218 home locations of participants in the Detroit-area NEXUS epidemiology study. Comparisons included examination of exposure distributions, spatial variability using maps coded by exposure group, Spearman’s and Kendal’s τ rank correlation coefficients, and concordance rates. While showing some agreement, simple categorical and proximity classifications (high diesel/low diesel traffic roads and distance from major roads) did not reflect the range and overlap of exposures seen in the other metrics. Information provided by the traffic density metric, defined as the number of kilometers traveled per day within a 300 m buffer around the home, was reasonably consistent with the more sophisticated metrics, although this metric does not provide information related to concentrations or temporal variability. Dispersion modeling provided this information, along with source apportionment results that separated concentrations from traffic emissions and other sources. At the NEXUS homes, the annual average and 24-h peak concentrations showed a high degree of spatial agreement. While there is broad agreement between several of the surrogate exposure metrics, including traffic density, emissions density and dispersion modeling, most of these alternatives still produced significantly different exposure classifications, suggesting the potential for exposure misclassification and the need for refined and validated exposure metrics.

The analyses also indicated the need for accurate geocoding of homes and roads given the spatial variability of pollutant levels near roads. Positional errors in the range of 30 to 50 m, and sometimes much more, should be anticipated using automated geocoding software and by positional errors in the representation of the road network. 

Dispersion modeling systems can provide exposure information relevant to on- and near-road environments, not only at homes, as demonstrated in the Detroit, but also at schools, parks, workplaces, commuting routes and other locations where people are exposed. While assembling the data and the computational demands for dispersion modeling of traffic emissions in large urban areas are non-trivial issues, future traffic-related health assessments, including epidemiological, risk and environmental justice studies, would benefit from such information. Further, this information can be used in hybrid models that simulate indoor exposures and time-activity behaviors, thus providing a refined estimate of air pollution exposure. At the community level, exposure assessments used in conjunction with transportation planning tools would advance policy initiatives aimed at mitigating traffic-related air pollutant exposures and health effects.

## Supplementary Files

Supplementary File 1
