# Supplementary material for: A Comparison of Exposure Metrics for Traffic-Related Air Pollutants: Application to Epidemiology Studies in Detroit, Michigan
Source: Int J Environ Res Public Health. 2014 Sep 15;11(9):9553–77. doi: 10.3390/ijerph110909553 (PMC4199035; doi:10.3390/ijerph110909553)
Supplement: Supplementary File 1 [file ijerph-11-09553-s001.pdf]

# A Comparison of Exposure Metrics for Traffic-Related Air Pollutants: Application to Epidemiology Studies in Detroit, Michigan

**Figure S1.** Distribution and maps of $NO_x$ emissions, defined as g/day for roads within 300 m of the home, coded by home group. High diesel roads are highlighted in yellow.

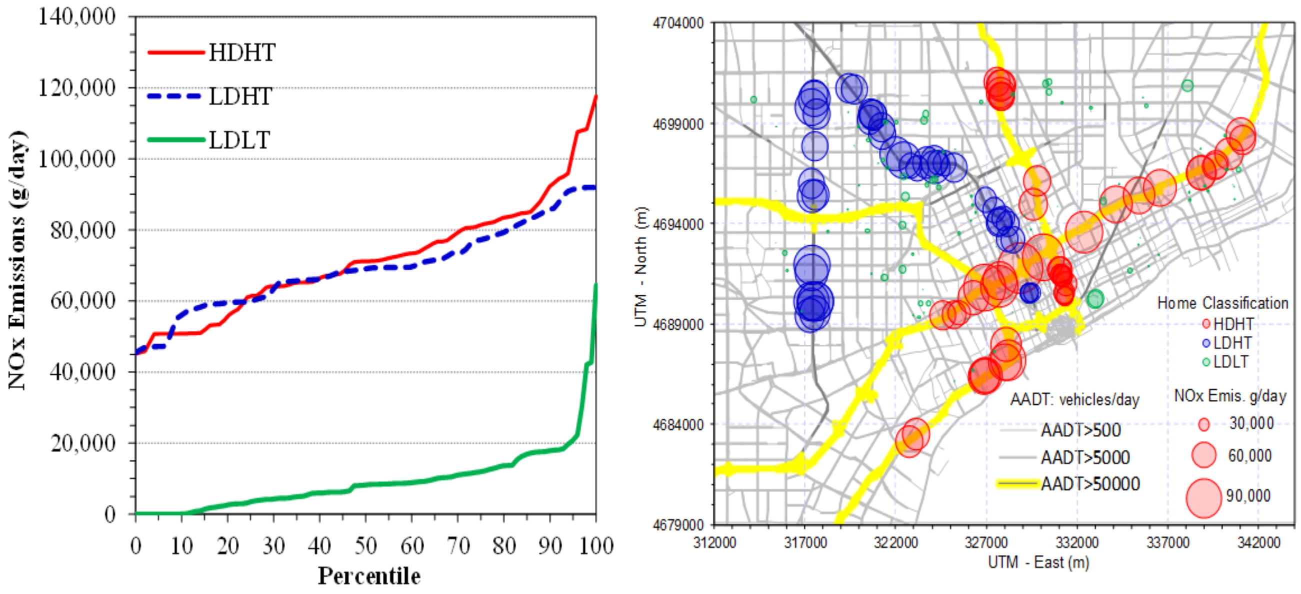


**Figure S2.** Distribution and maps of CO emissions, defined as g/day for roads within 300 m of the home, coded by home group. High diesel roads are highlighted in yellow.

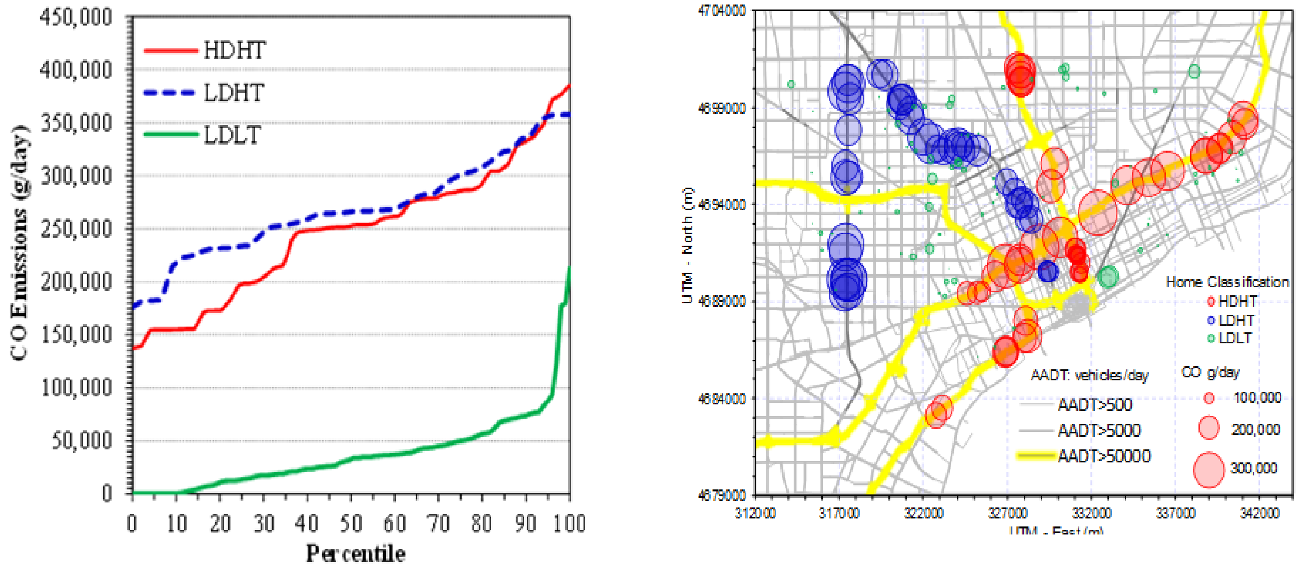

**Figure S3.** Distribution and maps of total annual average $PM_{2.5}$ predicted for 2010 from hybrid model. Each figure is coded by home group. High diesel roads are highlighted in yellow.

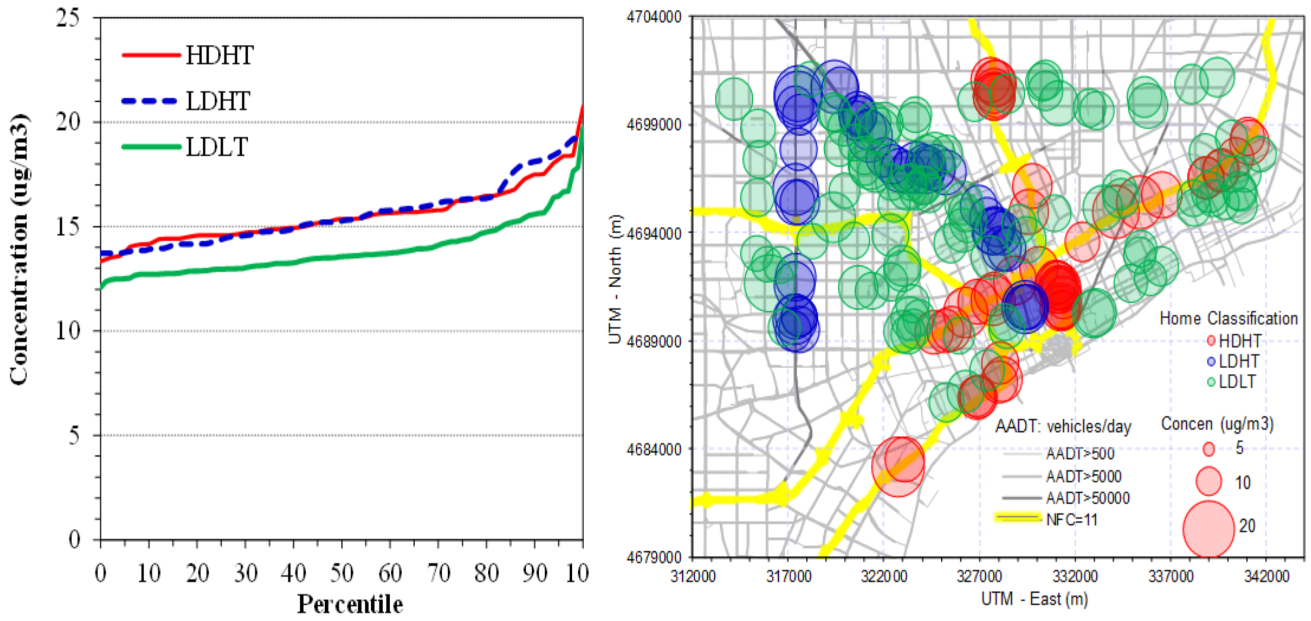


**Table S1.** Comparison of exposure metrics for NEXUS homes using rates of "severe" exposure misclassification in percent for tertiles and "thirds" groups. Shaded numbers show scores above 60% and 80%. Variable definitions as follows: Group = LDLT, LDHT and HDHT (assigned 1, 2, 3) categorical classification; Distance = m to nearest major highway; AADT = total traffic on nearest major highway; Lanes = number of lanes on nearest major highway; Diesel = diesel vehicles on nearest major highway; VKT = traffic density defined as vehicles-kilometers-traveled/day in 300 m buffer around home; PM Emis = $PM_{2.5}$ emissions in same buffer ; CO Emis = CO emissions in same buffer; PM Max = maximum 24-h $PM_{2.5}$ concentration; PM Ave = 2010 annual average $PM_{2.5}$ concentration. Sample size is $n$ = 218, except for comparisons involving AADT, Lanes, Diesel metrics where $n$ = 116 due to use of only high traffic homes.

| Type and Metric | Group (group) | Distance (m) | AADT (veh/day) | Lanes (no) | Diesel (veh/day) | VKT (km/day) | PM Emis (g/day) | CO Emis (g/day) | PM Max ($ug/m^3$) | PM Ave ($ug/m^3$) |
|---|---|---|---|---|---|---|---|---|---|---|
| **Severe disagreement among tertiles (percent)** | | | | | | | | | | |
| Distance | 51 | | | | | | | | | |
| AADT | 11 | 20 | | | | | | | | |
| Lanes | 31 | 7 | 19 | | | | | | | |
| Diesel | 20 | 20 | 4 | 16 | | | | | | |
| VKT | 47 | 1 | 11 | 9 | 22 | | | | | |
| PM Emis | 48 | 1 | 15 | 9 | 19 | 0 | | | | |
| CO Emis | 47 | 1 | 15 | 10 | 19 | 0 | | | | |
| PM Max | 46 | 2 | 15 | 8 | 16 | 1 | 1 | 1 | | |
| PM Ave | 53 | 3 | 16 | 3 | 18 | 1 | 1 | 1 | 0 | |

**Table S1.** *Cont.*

| Type and Metric | Group | Distance | AADT | Lanes | Diesel | VKT | PM Emis | CO Emis | PM Max | PM Ave |
|---|---|---|---|---|---|---|---|---|---|---|
| | (group) | (m) | (veh/day) | (no) | (veh/day) | (km/day) | (g/day) | (g/day) | (ug/m$^3$) | (ug/m$^3$) |
| **Severe disgreement with thirds (percent)** | | | | | | | | | | |
| Distance | 33 | | | | | | | | | |
| AADT | 66 | 34 | | | | | | | | |
| Lanes | 82 | 26 | 1 | | | | | | | |
| Diesel | 57 | 25 | 0 | 3 | | | | | | |
| VKT | 1 | 33 | 51 | 65 | 50 | | | | | |
| PM Emis | 0 | 33 | 46 | 61 | 44 | 0 | | | | |
| CO Emis | 1 | 33 | 52 | 65 | 51 | 0 | 0 | | | |
| PM Max | 8 | 42 | 44 | 56 | 43 | 3 | 1 | 3 | | |
| PM Ave | 4 | 46 | 50 | 62 | 48 | 6 | 2 | 7 | 0 | 0 |

© 2014 by the authors; licensee MDPI, Basel, Switzerland. This article is an open access article distributed under the terms and conditions of the Creative Commons Attribution license (http://creativecommons.org/licenses/by/3.0/).
